# Machine learning to optimize literature screening in medical guideline development

**DOI:** 10.1186/s13643-024-02590-5

**Published:** 2024-07-11

**Authors:** Wouter Harmsen, Janke de Groot, Albert Harkema, Ingeborg van Dusseldorp, Jonathan de Bruin, Sofie van den Brand, Rens van de Schoot

**Affiliations:** 1 Knowlegde Institute for the Federation of Medical Specialists, Utrecht, The Netherlands; 2 Department of Methodology and Statistics, Faculty of Social and Behavioral Sciences, Utrecht University, Utrecht, The Netherlands; 3 Department of Research and Data Management Services, Information Technology Services, Utrecht University, Utrecht, the Netherlands

**Keywords:** Guideline development, Active learning, Machine learning, Systematic reviewing

## Abstract

**Objectives:**

In a time of exponential growth of new evidence supporting clinical decision-making, combined with a labor-intensive process of selecting this evidence, methods are needed to speed up current processes to keep medical guidelines up-to-date. This study evaluated the performance and feasibility of active learning to support the selection of relevant publications within medical guideline development and to study the role of noisy labels.

**Design:**

We used a mixed-methods design. Two independent clinicians’ manual process of literature selection was evaluated for 14 searches. This was followed by a series of simulations investigating the performance of random reading versus using screening prioritization based on active learning. We identified hard-to-find papers and checked the labels in a reflective dialogue.

**Main outcome measures:**

Inter-rater reliability was assessed using Cohen’s Kappa (*ĸ*). To evaluate the performance of active learning, we used the Work Saved over Sampling at 95% recall (WSS@95) and percentage Relevant Records Found at reading only 10% of the total number of records (RRF@10). We used the average time to discovery (ATD) to detect records with potentially noisy labels. Finally, the accuracy of labeling was discussed in a reflective dialogue with guideline developers.

**Results:**

Mean *ĸ* for manual title-abstract selection by clinicians was 0.50 and varied between − 0.01 and 0.87 based on 5.021 abstracts. WSS@95 ranged from 50.15% (SD = 17.7) based on selection by clinicians to 69.24% (SD = 11.5) based on the selection by research methodologist up to 75.76% (SD = 12.2) based on the final full-text inclusion. A similar pattern was seen for RRF@10, ranging from 48.31% (SD = 23.3) to 62.8% (SD = 21.20) and 65.58% (SD = 23.25). The performance of active learning deteriorates with higher noise. Compared with the final full-text selection, the selection made by clinicians or research methodologists deteriorated WSS@95 by 25.61% and 6.25%, respectively.

**Conclusion:**

While active machine learning tools can accelerate the process of literature screening within guideline development, they can only work as well as the input given by human raters. Noisy labels make noisy machine learning.

## Introduction

Producing and updating trustworthy medical guidelines is a deliberative process that requires a substantial investment of time and resources [1]. In the Netherlands, medical guidelines in specialist medical care are being developed and revised in co-production between clinicians and guideline methodologists. There are over 650 medical specialists’ guidelines in the Netherlands, answering approximately 12,000 clinical questions. An essential element in guideline development is a systematic synthesis of the evidence. This systematic appraisal includes the formulation of clinical questions, selection of relevant sources, a systematic literature review, grading the certainty of the body of evidence using GRADE [2], and finally, translating the evidence into recommendations for clinical practice [3].

Evidence synthesis starts with translating a clinical question into a research question. Hereafter, a medical information specialist systematically searches literature in different databases. Then, literature screening is performed independently by two clinicians who label relevant publications based on inclusion and exclusion criteria in the title or abstract. Once the relevant publications have been selected, a guideline methodologist with more experience in systematically selecting relevant publications from large datasets supports further title-abstract selection, assessing the methodological quality of the selected papers. Literature screening is time-consuming, with an estimated 0.9 and 7 min per reference per reviewer for abstract and full-text screening, respectively. Since a single literature search can easily result in hundreds to thousands of publications, this can add up to 100–1000 min of selection based on title and abstract and even more so for full-text selection [4]. In an era of exponential growth of new evidence, combined with a labor-intensive process, there is a need for methods to speed up current processes to keep medical guidelines up-to-date.

The rapidly evolving field of artificial intelligence *(AI)* has allowed the development of tools that assist in finding relevant texts for search tasks [5–16]. A well-established approach to increasing the efficiency of title and abstract screening is screening prioritization [17] via *active learning* [18]. With machine learning models, relevance scores for each publication can be computed. Then, assessors label titles and abstracts (relevant versus irrelevant) for each most relevant record, and the model iteratively updates its predictions based on the given labels and prioritizes articles that are most likely to be relevant. Active learning is found to be extremely effective for systematic reviewing (see for a systematic review [19]).

 Implementing active learning could save a tremendous amount of work and time and may open a new window of opportunity in the context of evidence-based guideline development. However, active learning works under the strong assumption that given labels are correct [20]. While research with experienced reviewers may be straightforward, working with clinical questions and clinicians in the daily practice of guideline development may be more complex. Most clinicians are not experienced with title-abstract selection and often perform this task in addition to their daily work in the clinic. With large numbers of abstracts and limited time, clinicians can become distracted or fatigued, introducing variability in the quality of their annotations. This variability in human performance may hinder the applicability of active learning in guideline development. Given the potential of active learning and the more complex context of guideline development, this practice-based study aimed to evaluate the performance and feasibility of active learning to support literature screening within the context of guideline development.

We aim to evaluate the added value of using active learning and the impact of noisy labels during three stages of the review process: (1) title abstract selection by clinicians, (2) additional title abstract selection by experienced research methodologists, and (3) final full-text inclusions after expert consensus. In what follows, we present the 14 datasets used and the workflow for manual literature screening in guideline development and introduce the setup of active learning. This is followed by a simulation study mimicking the screening process for the 14 clinical questions, comparing the performance of literature screening using active learning versus manual selection in terms of work saved over sampling and the average time to discovery for identifying hard-to-find papers potentially having a noisy label [21, 22]. We then present the results of the discussion of the hard-to-find papers in a reflective dialogue with the research methodologists and evaluate reasons that facilitate or hamper the performance of active learning.

## Methods

### Datasets

We selected 14 clinical questions from recently published clinical guidelines containing manually labeled datasets, providing a wide range of types and complexity of clinical questions; see Table 1. The datasets were derived from different guidelines published between 2019 and 2021, covering different types of questions, e.g., diagnostic, prognostic, and intervention types of questions. In order to be sure that the guidelines had been authorized and thus finished, we selected those that are openly published on the Dutch Medical Guideline Database [Richtlijnendatabase.nl]. Per clinical question, two clinicians independently labeled title-abstracts using prespecified inclusion and exclusion criteria. The datasets contained (at least) the papers’ title and abstract plus the labels relevant/irrelevant for each annotator (clinician and research methodologist) and the column with the final inclusion. Duplicates and papers with missing abstracts were removed from the dataset. All datasets can be found on the Open Science Framework page of the project: https://osf.io/vt3n4/.

**Table 1:** Descriptive characteristics of the purposefully selected datasets (*n* = 14)

#	Guideline topic	Medical specialty	Type of question	*N*	Screening time (min)	*К*
1	Radial fractures approach	General surgery	Intervention	195	225	0.31
2	Radial fractures closed reduction	General surgery	Prognostic	277	294	0.55
3	Hallux valgus prognostic	Orthopedic surgery	Prognostic	640	327	0.64
4	Head and neck cancer bone	Otolaryngology	Diagnostic	311	253	0.87
5	Head and neck cancer imaging	Otolaryngology	Diagnostic	56	72	0.61
6	Obstetric emergency training	Obstetrics	Intervention	188	275	0.61
7	Post-intensive care treatment	Rehabilitation	Intervention	435	388	0.05
8	Pregnancy medication	Obstetrics	Intervention	428	243	0.66
9	Shoulder replacement diagnostic	Radiology	Intervention	215	123	0.59
10	Shoulder replacement surgery	Orthopedic surgery	Intervention	335	270	0.57
11	Shoulder dystocia positioning	Gynecology	Diagnostic	342	366	0.49
12	Shoulder dystocia recurrence	Gynecology	Intervention	397	172	-0,01
13	Total knee replacement	Orthopedic surgery	Intervention	480	262	0.55
14	Vascular access	General surgery	Intervention	722	496	0.51

### Manual screening

To evaluate inter-rater reliability for the manual literature screening, we used Cohen’s Kappa index measure [23]. Cohen’s Kappa gives relevant information on the amount of consensus among different raters with higher scores indicating better interrater agreement.

### Simulation

Utilizing the labeled datasets, we conducted various simulation studies to explore the intricacies of model performance. Each simulation emulates the screening process using a specific model, guiding the algorithm through the dataset according to predefined strategies using a specific active learning model. The performance is typically evaluated by randomly screening a labeled dataset. This setup allows the simulation to replicate the screening process, akin to a researcher conducting AI-assisted screening, thereby providing a realistic representation of how the model would perform in practical applications. These simulations are distinct from traditional statistical simulation studies in several key aspects. Firstly, the primary objective of our simulations is to evaluate the efficacy of AI algorithms in literature screening. This is in contrast to typical statistical simulations, which often focus on assessing theoretical statistical properties such as power, bias, or variance under various hypothetical scenarios. Also, we make use of real-world, labeled datasets, diverging from the standard practice in statistical simulations that frequently rely on hypothetical or synthetically generated data. This use of actual data from literature ensures a more practical and application-oriented assessment of the model’s performance.

The simulations were conducted with the command line interface of ASReview version v0.16 [24]. ASReview has been proven to be a valid tool for the selection of literature in numerous studies [15, 21, 25–33]. We used Naïve Bayes as the classifier for the simulation study with TF-IDF as the feature extraction technique.

In our study, each dataset underwent simulations targeting different sets of relevant records as defined by three groups: (1) clinicians, (2) a combination of clinicians and research methodologists, and (3) the final inclusion decisions. We included one relevant record as a prior inclusion for each simulation’s training data, along with ten randomly selected irrelevant records. We conducted multiple runs for each dataset to mitigate the potential bias introduced by the starting paper in the model’s first iteration, varying the relevant paper used in the initial training. The outcomes are averaged over these runs to ensure a balanced assessment. Consistency was maintained within each run of a dataset by using the same ten irrelevant records.

We analyzed the model performance of active learning by calculating the following three outcome measures: the Work Saved over Sampling (WSS), which indicates the reduction in publications needed to be screened at a given level of recall [17]. The WSS is typically measured at a recall level of 95%; WSS@95 reflects the amount of work saved by using active learning at the cost of failing to identify 5% of relevant publications. Note that humans typically misclassify about 10% [34]. Secondly, we computed the metric Relevant Records Found (RRF), which represents the proportion of relevant publications that are found after screening a prespecified percentage of all publications. Here, we calculated RRF@10, which represents the percentage of relevant publications found after screening only 10% of all publications. Thirdly, we calculate the average time to discovery (ATD) [21] and the fraction of non-reviewed relevant publications during the review (except the relevant publications in the initial dataset). The ATD is an indicator of the performance throughout the entire screening process instead of performance at some arbitrary cutoff value. The ATD is computed by taking the average of the time to discovery (TD) of all relevant publications. The TD for a given relevant publication *i* is computed as the fraction of publications needed to screen to detect *i*. We used this metric to identify hard-to-find papers potentially having a noisy label.

We also plotted recall curves to visualize model performance throughout the entire simulation. Recall curves give information in two directions; they display the number of publications that need to be screened and the number of relevant publications.

All scripts to reproduce the simulations are available at 10.5281/zenodo.5031390

### Reflective dialogue

In order to better understand the differences in performance across the different datasets, we organized a reflective dialogue. In a two 3.5-h session, seven research methodologists who initially labeled the datasets critically appraised the quality of the labeled datasets in light of the performance. Specifically, we wanted to know why some publications were found very easy and others more difficult to zoom in on the hard-to-find papers to identify possible noisy labels.

## Results

### Manual screening

The selected datasets encompass seven different medical fields, addressing intervention, diagnostic, and prognostic types of questions. Table 1 details the datasets by guideline topic, medical specialty, type of question, number of abstracts screened, minute screening time, and Cohen’s Kappa (*ĸ*) for interrater agreement.

In our study, twenty-four clinicians independently screened a total of 5021 abstracts across all datasets. From these, they identified 339 potentially relevant publications, which required 3766 min of screening time. The mean *ĸ* for interrater agreement across all datasets was 0.50, with individual values ranging from − 0.01 to 0.87, as detailed in Table 1 for each specific dataset.

Out of the 339 publications initially identified as relevant by clinicians, the research methodologists excluded 166 (49%) due to methodological concerns. A further 45 (13.3%) were excluded after full-text review, leaving 128 publications for final full-text inclusion. Table 1 also reflects these figures, presenting a breakdown of the initial abstract screening results for each of the 14 purposefully selected datasets, including the specific medical specialty and question type they pertain to.

### Simulation

The simulation study results are summarized in Table 2, presenting a comprehensive analysis of datasets labeled by clinicians and research methodologists following full-text selection.

**Table 2:** Results from simulation analyses for datasets labeled by clinicians, research methodologists, and full-text selection

#	*N*	Select_Cl_	Select_Ex_	Select_FT_	WSS95_Cl_	WSS95_Ex_	WSS95_FT_	RRF10_Cl_	RRF10_Ex_	RRF10_FT_
1	195	11	6	5	32.31 (6.37)	57.70 (4.80)	61.41 (2.14)	29.09 (8.31)	30.00 (16.73)	20.00 (11.18)
2	277	8	4	4	43.33 (5.47)	59.40 (6.82)	62.31 (9.15)	28.57 (13.23)	25.00 (16.67)	33.33 (27.22)
3	640	20	14	12	55.76 (2.54)	73.55 (1.54)	77.40 (2.45)	43.16 (5.56)	52.75 (7.90)	62.88 (10.59)
4	311	34	20	11	73.15 (1.43)	72.98 (2.48)	78.12 (4.10)	66.22 (4.10)	71.32 (11.39)	73.64 (9.24)
5	56	18	9	8	48.89 (0.00)	70.12 (3.35)	70.28 (3.13)	28.10 (2.52)	45.83 (12.50)	41.07 (5.05)
6	188	18	12	7	40.33 (2.47)	45.34 (8.99)	86.76 (1.78)	47.06 (6.69)	40.15 (11.92)	78.57 (15.85)
7	435	109	22	6	32.70 (1.25)	66.10 (1.08)	64.19 (11.75)	25.63 (3.31)	62.55 (5.93)	46.67 (20.66)
8	428	45	45	45	66.42 (1.26)	66.34 (1.08)	66.92 (1.23)	60.45 (5.34)	61.26 (5.52)	60.86 (5.31)
9	342	3	1^a^	1^a^	97.99 (0.70)	NA	NA	100.00 (0)	NA	NA
10	397	6	4	4	74.78 (2.53)	93.78 (0.87)	93.13 (0.15)	63.33 (8.16)	100.00 (0)	100.00 (0)
11	218	6	5	4	79.55 (1.82)	82.80 (0.43)	79.95 (2.51)	46.67 (20.66)	40.00 (13.69)	33.33 (0)
12	335	5	5	4	61.30 (14.84)	61.05 (14.20)	96.68 (0.92)	65.00 (22.36)	60.00 (33.54)	100.00 (0)
13	480	35	16	9	65.09 (4.03)	73.12 (4.00)	95.76 (0.34)	78.74 (10.14)	89.17 (16.67)	100.00 (0)
14	772	21	10	8	34.84 (16.34)	95.72 (0.32)	96.27 (0.48)	85.95 (19.72)	100.00 (0)	100.00 (0)
**Total**	5074	339	173	128	50.15 (17.14)	69.24 (11.51)	75.76 (12.16)	48.31 (23.32)	62.78 (21.20)	65.58 (23.25)

^a^A simulation study with only 1 relevant paper cannot be executed because it needs at least 1 relevant record as training data and 1 relevant record as target paper to detect

Select_Cl, Ex, FT_ = number of records included by the clinician, research methodologist, and full-text selection

WSS95_Cl, Ex, FT_ = Work Saved over Sampling measured at a recall level of 95% for dataset labeled by the clinician, research methodologist, and full-text selection

RRF10_Cl, Ex, FT_ = Relevant References Found after screening 10% of all publications (RRF10) for dataset labeled by the clinician, research methodologist, and full-text selection

It showed that the Work Saved over Sampling (WSS@95) was lowest for clinicians and ranged from 32.31 to 97.99%, with a mean of 50.15% (SD = 17.74); followed by the research methodologist, it ranged from 45.34 to 95.7%, with a mean of 69.24% (SD = 11.51); and simulating the full-text inclusions resulted in the highest WSS@95 that ranged from 61.41 to 96.68% (0.92), with a mean of 75.76% (SD = 12.16).

A similar pattern emerged for RRF@10 which, for clinicians, ranged from 28.10 to 100%, with a mean of 48.31% (SD = 23.32); for the research methodologist, it ranged from 25.00 to 100%, with a mean of 62.78% (SD = 21.20); and simulating full-text inclusions gave an RRF@10 that ranged from 20.00 to 100% (0.92), with a mean of 65.58% (SD = 23.25). ATD Ranged from screening 20 to 62 abstracts.

**Fig. 1:**
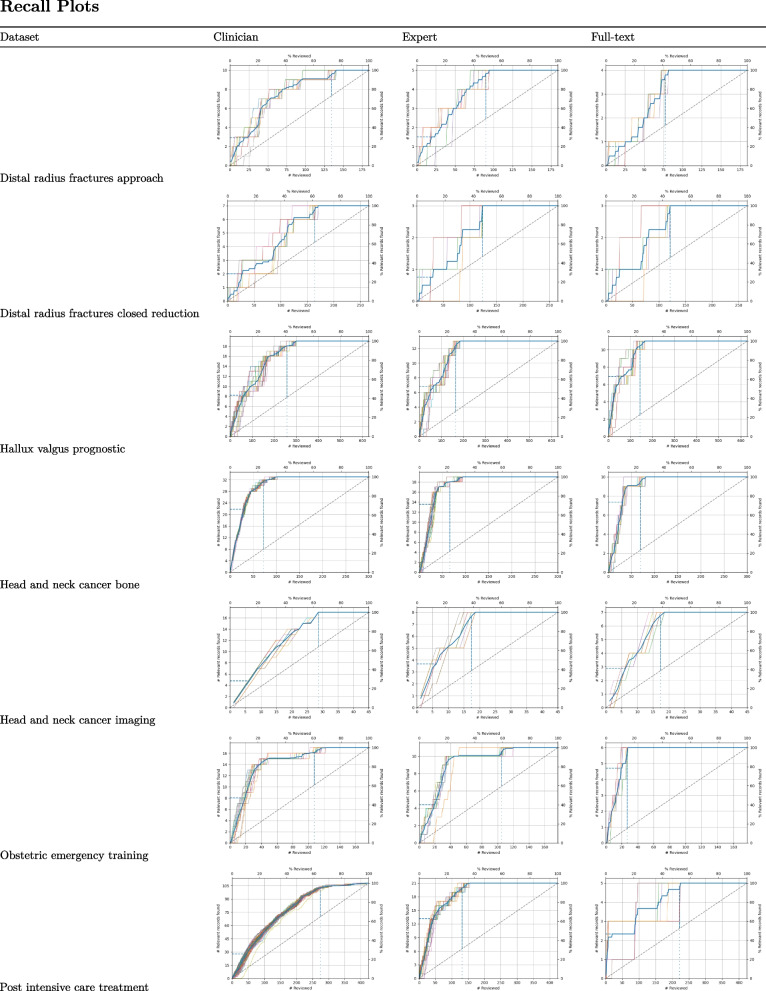

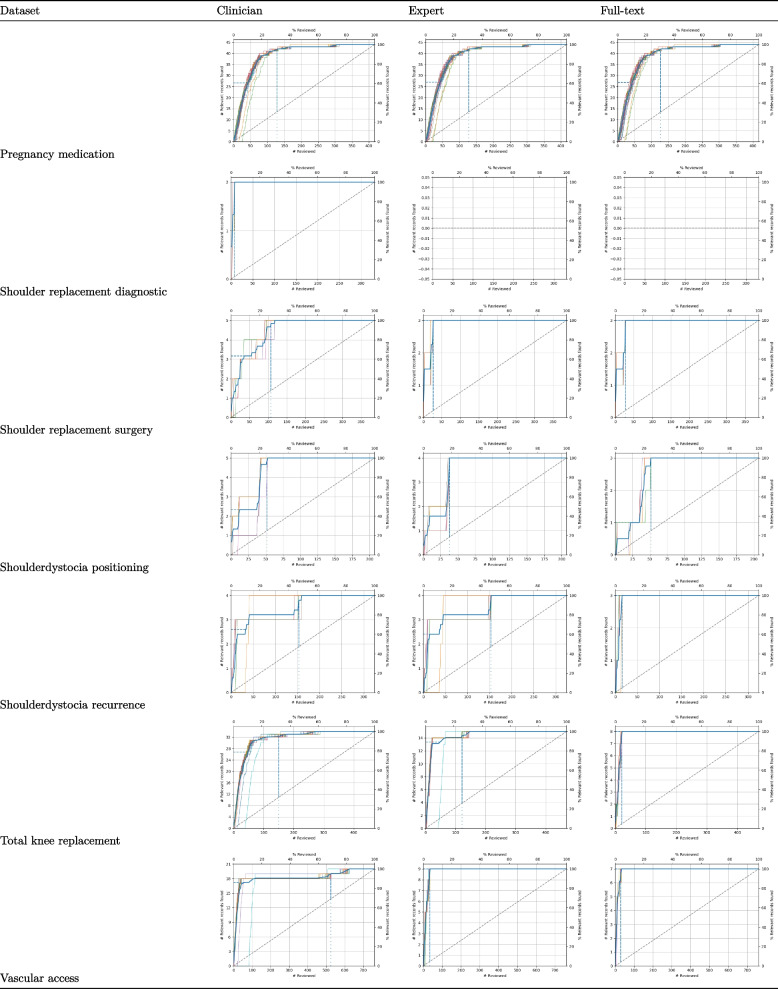
Recall plots of the simulated datasets. The first row indicates the number of relevant records found for each simulation run as displayed as a function of the number of records screened for each of the three levels (clinician, guideline methodologist, final decision). The vertical line indicates when 95% of the relevant records have been found. The *Y*-axis presents the number of relevant papers minus one paper selected for training data. Note that the simulation for the dataset *Shoulder_replacement_diagnostic* shows no recall lines because only one relevant paper was included, and at least two relevant records are needed for a simulation study

Figure 1 presents recall curves for *all* simulations, and as can observed, the recall curves differ across datasets but always outperform randomly reading the records, which is the standard approach.

To illustrate these results in a more tangible context, we discuss one dataset in detail: *Distal_radius_fractures_approach*. Out of the 195 records identified in the search, 11 (5.64%) were indicated as relevant by the clinicians, 6 (3.08%) by the guideline methodologist, and, ultimately, only 5 (2.56%) were included in the final protocol. Zooming in on WSS@95 for full-text inclusions, on average, after screening 43% of the records (*n* = 83), all records (5 out of 5) would have been found. If one screened records in a random order, at this point, one would have found 3 of the relevant records, and finding 5 of the relevant records would take, on average, 186 records. In other words, the time that can be saved using active learning expressed as the percentage of records that do not have to be screened is 61% (sd = 5.43), while still identifying 95% of the relevant records. The RRF@10 is 20% (sd = 11.18), meaning that after screening 10% of records, 20% of the relevant records have been identified.

### Reflective dialogue

During these sessions, we reflected on the current progress of selecting relevant publications and how this affected some of the difficulties in active learning. The discussion during the reflective dialogue revealed that almost half (= 49%) of the selected publications by the clinicians did *not* meet the predefined inclusion criteria, e.g., PICO criteria or study design, and were, therefore, later re-labeled as irrelevant by the research methodologists.

In this reflective dialogue, we also discussed the performance of active learning in specific datasets. While for some active learning seemed to be hindered by incorrect inclusion by the clinicians, in other samples, active learning had difficulty due to the structure of the abstracts from studies other than RCTs. For example, the recall plots for the dataset *Distal_radius_fractures_approach* showed that the clinicians identified five papers as relevant, which were later deemed irrelevant by the guideline methodologists. Methodologists mentioned how clinicians would often include studies for other reasons (interesting to read, not daring to exclude, or not knowing the exact inclusion criteria). This led to the mention of the “noisy labels” for inclusions that should not have been included in the first place. In the current manual process, these are excluded by the methodologist, which takes extra time. For other datasets (i.e., *Shoulder_replacement_surgery*, *Total_knee_replacement*, and *Shoulder_dystocia_positioning*), active learning seems to have difficulty in finding systematic reviews and observational studies compared to randomized control trials. As discussed, this may be inherent to the way the abstracts are structured, e.g., RCTs often describe a strict comparison, while this may be less evident for systematic reviews and observational studies.

## Discussion

The purpose of this practice-based study was to evaluate the performance and feasibility of active learning to support the selection of relevant publications within the context of guideline development. Although ASReview has been proven to be a valid tool for the selection of literature in numerous studies [15, 21, 25–33], none tested the performance on medical guidelines. We evaluated the performance of active learning on labeled datasets from 14 clinical questions across the three different stages of the review process. The simulations show a considerable variation in the reduction of papers needed to be screened (13–98%). The variation is caused by the clearness and coherence of the abstracts, the specificity of the inclusion and exclusion criteria, and whether the information for full-text inclusion is actually present in the abstract. On average, however, when active learning models were used, the WSS@95 was 50% for the screening done by clinicians. After additional assessment by an experienced research methodologist, the average WSS@95 increased to 69%, with a further increase to 75% after final full-text inclusion. This means that the performance of active learning increases with more accurate title-abstract labeling, which underlines the importance of strict inclusion and exclusion criteria.

The results of the reflective dialogue emphasize that in the current way of selection, inclusion and exclusion by clinicians in guideline development is not always as straightforward as in systematic reviews by researchers. Our results align with the hypothesis that active learning works under the strong assumption that given labels are correct [20]. During our reflective dialogue session, the notion of “noisy labels” was introduced for the initial screening process. This notion was confirmed in the low to moderate interrater reliability of the manual title-abstract screening, with an average kappa of 0.5 in line with other recent findings [35]. Therefore, our main conclusion is that active learning models can speed up the process of literature screening within guideline development but, at the same time, assume correct labels of inclusion and exclusion, as our data showed that performance was dependent on the quality of the annotations.

Our next question, therefore, was to find the “noise” in the manual screening process. Some interesting themes emerged when looking at the differences between the selections made by the clinicians and the professional guideline developers. Guideline methodologists realized that clinicians often include publications based on the PICO criteria and out of personal interest or fear of leaving out important data. Indeed, when re-examined, many articles did not fall within the PICO criteria or the predefined criteria regarding methodological concerns (e.g., RCT vs. case–control studies or cohort studies). On average, there was a 49% drop in inclusion when the guideline methodologist re-evaluated the original inclusion made by the clinicians.

A question of interest for future study is when to trust that all relevant literature on the topic has been retrieved based on our results and others. In this study, we plotted recall curves to visualize active learning performance and organized discussion meetings to reason why some publications were more difficult to find. Looking at the examples, this often happened when the search had followed a slightly different process. In the current workflow, due to limited resources, pragmatic choices are being made not to include all individual studies when a recent systematic review is available. For active learning models, it takes time to “learn” this adapted (non-logical) strategy. For instance, plateaus occur in some of the recall plots, and after a series of irrelevant records have been identified, a new relevant record was found. Interestingly, when time is saved by working with active learning tools, these pragmatic choices might not be necessary anymore and may lead to a much larger and more complete set of inclusions than the manual workflow.

### Strengths and weaknesses

While an obvious weakness concerns the number of datasets included, in this study, we did not cover all types of clinical questions, and our findings are mainly based on intervention types of questions. On the other hand, a strength of this study is that we evaluated the daily practice of guideline development using real-world data from previously developed guidelines. While studies are reporting on tools implementing active learning in systematic reviews, there is little evidence of implementing such tools in daily practice [36–38]. Our “real world” data provided us with new challenges not seen before because it is frequently tested in research settings without going back to the initial screeners, leaving out more pragmatic and human-interest choices that influence literature screening.

This type of practice-based study has shown potential ways to use and improve current practice. In our sample, active learning detected the most relevant studies with a significant reduction in the number of abstracts that needed to be screened. The system performed better when the inclusion and exclusion criteria were adhered to more strictly. The findings brought us to look at the workflow needing more attention to guide the clinicians in the systematic selection of papers. This is beneficial not only when using software like ASReview, where the principle of “quality in, quality out” seems to apply, but also when using the manual selection of papers. After abstract screening, almost half of the inclusions were incorrect, which is higher than the error rates reported in systematic reviews, with a mean error rate of nearly 11% over 25 systematic reviews [34]. Methods to improve literature selection have been described [39, 40] and include recommendations to include reflection and group discussion, resulting in a more iterative process, practical tips like taking regular breaks and coding in small batches at a time to prevent fatigue, but also setting up unambiguous inclusion criteria and adjusting the codebook during the process if needed. While the inclusion of two independent reviewers is often assumed to be the best way to reduce bias, these authors also advise regularly assessing interrater reliability as part of reflective and learning practice.

We also defined some remaining questions for future research. As described above, in guideline development, research questions do not always yield prior inclusion papers, while the performance of active learning partially depends on at least one relevant starting paper to learn from. A possible solution that needs to be explored might be to start with a dummy abstract containing all relevant elements from the PICO. At the same time, we need more samples of research questions in clinical guidelines to further evaluate the use of AI tools in different questions and contexts. In this study, we evaluated a limited set of retrospective data using one active learning algorithm, and future studies could explore more datasets, different active learning algorithms, or different tools in different phases of the process of guideline development to evaluate further the human–machine interaction and how this affects the process of guideline development.

## Conclusions

This study shows a 50–75% reduction in abstracts that needed to be screened to find and select all relevant literature for inclusion in medical guidelines when using active learning models. At the same time, this study also shows the importance of the quality of the human input as it directly relates to the performance of active machine learning. The next step would be to evaluate how to apply active learning in the workflow of guideline development, how to improve human input, and what it means for both the timeframe to develop new recommendations and the transparency and quality of these evidence-based recommendations.

## AI statement

During the research and the creation of this research paper, AI-based tools like Grammarly, for grammar and language mechanics and OpenAI’s ChatGPT, for code creation and content review, were utilized. These were supplementary aids, and all final interpretations and content decisions were the sole responsibility of the authors.

## Data Availability

All scripts that were used during this study, including preprocessing, analyzing, and simulation scripts for results, figures, and tables published in this paper, can be found on the GitHub page of the project: 10.5281/zenodo.5031390. The 14 systematic review datasets are openly available on the Open Science Framework https://osf.io/vt3n4/
